# Natural Products for Chronic Diseases: A Ray of Hope

**DOI:** 10.3390/molecules27175573

**Published:** 2022-08-30

**Authors:** Syed Shams ul Hassan, Mohamed M. Abdel-Daim, Tapan Behl, Simona Bungau

**Affiliations:** 1 Shanghai Key Laboratory for Molecular Engineering of Chiral Drugs, School of Pharmacy, Shanghai Jiao Tong University, Shanghai 200240, China; 2 Department of Natural Product Chemistry, School of Pharmacy, Shanghai Jiao Tong University, Shanghai 200240, China; 3 Department of Pharmaceutical Sciences, Pharmacy Program, Batterjee Medical College, P.O. Box 6231, Jeddah 21442, Saudi Arabia; 4 Pharmacology Department, Faculty of Veterinary Medicine, Suez Canal University, Ismailia 41522, Egypt; 5 School of Health Sciences and Technology, University of Petroleum and Energy Studies, Dehradun 248007, Uttarakhand, India; 6 Department of Pharmacy, Faculty of Medicine and Pharmacy, University of Oradea, 410028 Oradea, Romania; 7 Doctoral School of Biomedical Sciences, University of Oradea, 410087 Oradea, Romania

This Special Issue includes many high advanced quality papers that focus on natural products with their potent pharmacological potential targeting various areas of diseases. The papers in this Special Issue present new insights into natural products with potent anticancer, anti-inflammatory, antioxidant, anti-bacterial, analgesic, anti-diabetic, and enzyme inhibitory activities.

Secondary metabolites from nature, predominantly plants, are still a research hotspot because of their promising novel scaffolds against chronic diseases. Plant-derived bioactive compounds were proved to have promising anticancer activities. Recently, many researchers have driven their research interest toward plants to evaluate the use of plant-derived bioactive compounds against different kinds of cancer. Hassan and his team [1] evaluated one guaiane-type sesquiterpene dimer vieloplain F from Xylopia Vielana species against melanoma by targeting B-Raf kinase. The results indicated that vieloplain F has good anticancer activity against melanoma by displaying a higher binding energy of −11.8 kcal/mol against B-Raf protein compared to the FDA-approved drug vemurafenib. Further MD simulations and MM-GBSA showed that vieloplain F had the most remarkable binding propensity to active site residues. In addition, the absorption, distribution, metabolism, excretion, and toxicity (ADMET) profile of the FDA-approved medicine vemurafenib was hepatotoxic, cytochrome-inhibiting, and non-cardiotoxic compared to vieloplain F, which at this moment has been selected for further investigation due to its potential effects against melanoma. Majid et al. [2] isolated a new triterpenoid nummularic acid (NA) from Ipomoea batatas and evaluated its anticancer activity against prostate cancer (PCa). The results showed that significant (*p* < 0.05 and *p* < 0.01) time and dose-dependent reductions in the proliferation of PCa cells, reduced migration, invasion, and an increased apoptotic cell population were recorded after NA treatment (3–50 µM). Further profound mechanistic studies revealed that NA treatment considerably increased the cleavage of caspases and downstream PARP, upregulated BAX and P53, and downregulated BCL-2 and NF-κB, inducing apoptosis in PCa cells. Khan et al. [3] evaluated the effects of DL-propargylglycine (PAG, inhibitor of CSE), aminooxy acetic acid (AOAA, inhibitor of CBS), and L-aspartic acid (L-Asp, inhibitor of 3-MPST) against breast cancer (BC) by determining the role of endogenous H_2_S in the growth of BC by performing in vitro and in vivo experiments. The results showed that the combined dose (PAG + AOAA + L-Asp) group showed exclusive inhibitory effects against BC cells’ viability, proliferation, migration, and invasion compared to the control group. Further, treated cells exhibited increased apoptosis and a reduced level of phospho (p)-extracellular signal-regulated protein kinases such as p-AKT, p-PI3K, and p-mTOR. Moreover, the combined group exhibited potent inhibitory effects on the growth of BC xenograft tumors in nude mice without apparent toxicity.

Natural products have a broad pharmacological spectrum because of their complex scaffolds. Huneif et al. [4] isolated two compounds from wild strawberries and evaluated their anti-diabetic and antioxidant activity. The results showed that both compounds have good anti-diabetic activity against α-glucosidase, α-amylase, and antioxidant activity against DPPH free radicals. Al-Joufi et al. [5] evaluated the anti-diabetic, antioxidant, and anti-microbial potential of the Anabasis articulata plant. The results showed that different extracts (methanolic and n-hexane) displayed remarkable anti-diabetic activity against α-glucosidase, α-amylase, antioxidant activity against DPPH free radicals and anti-microbial activity against *Shigella dysentery* (*S. dynasties*), *Escherichia coli* (*E. coli*), and *Salmonella typhi* (*S. typhi*). Ahmed et al. [6] evaluated the vegetable plant Pleurospermum candollei by investigating its phytochemical profile and biological activities such as antioxidant, anti-bacterial, thrombolytic and enzyme inhibitory characteristics (tyrosinase, α-amylase, and α-glucosidase). The results displayed that methanolic and n-hexane extracts showed remarkable pharmacological activities in terms of antioxidant, anti-bacterial, thrombolytic and enzyme inhibitory characteristics. In addition, pure compounds also displayed good docking results against targeted proteins. Mahmood et al. [7] evaluated the anti-inflammatory, analgesic and antioxidant capacity of New (2*S*,3*S*)-2-(4-isopropylbenzyl)-2-methyl-4-nitro-3-phenylbutanals. The results revealed that two compounds have potent anti-inflammatory activity in vitro against COX ½ and 5-LOX, antioxidant activity against DPPH free radicals and in vivo analgesic activity. Faheem et al. [8] investigated the effects of natural compounds, berbamine, bergapten, and carveol on paclitaxel-associated neuroinflammatory pain. The results revealed that all the compounds attenuated thermal hypersensitivity and increased the threshold for pain sensation. The compounds also increased the protective glutathione (GSH) and glutathione S-transferase (GST) levels in the sciatic nerve and spinal cord while lowering inducible nitric oxide synthase (iNOS) and lipid peroxidase (LPO). Furthermore, the compounds also inhibited cyclooxygenase-2 (COX-2), tumor necrosis factor-alpha (TNF-α), and nuclear factor kappa B (NF-κb) overexpression.

Glensk et al. [9] isolated bioactive compounds echimidine and its C-7 isomers from Echium plantagineum L. and evaluated their hepatotoxic effect on rat hepatocytes. The results revealed that the compounds at 3 to 300 µg/mL caused the concentration-dependent inhibition of hepatocyte viability, with mean IC_50_ values ranging from 9.26 to 14.14 µg/mL. This study revealed that under standard HPLC acidic conditions, echimidine co-elutes with its isomers, echihumiline and to a lesser degree with hydroxy myoscorpine, obscuring the actual alkaloidal composition, which may have implications for human toxicity. Khan et al. [10] evaluated the effects of shrimp peptide hydrolysate on intestinal microbiota restoration and immune modulation in cyclophosphamide-treated mice. The results showed that shrimp peptide hydrolysate significantly restored goblet cells and intestinal mucosa integrity, modulated the immune system, and increased the relative expression of mRNA and the tight-junction associated proteins occludin, Zo-1, claudin-1, and mucin-2).

Marine drugs possess an undoubtedly diverse range of sources as they are distributed over 70% of the earth’s surface, possess a wide range of variations in structure and present a promising target in the discovery of newer and better treatment approaches. In the past seven decades, many structurally diverse drug products and their secondary metabolites have been isolated from marine sources which have successfully presented an exceptional potential in the treatment of various diseases ranging from acute to chronic conditions. Hence, Bhatia et al. [11] highlighted the significant role of marine-derived drugs in the management of chronic diseases such as diabetes, cancer, cardiovascular and neurodegenerative disorders.

Principally, the Special Issue “Natural Products for Chronic Diseases: A Ray of Hope” provides a current perspective of the natural products from the marine and terrestrial area and the rapidly developing research area, as evident from the resistance to the available drugs and wide variety of chronic diseases. Considering the challenges in this exciting field of natural products drug discovery, this issue not only complements our knowledge on bioactive compounds but also may uncover some novel ideas and motivation for the further investigation of various prospective biologically active compounds impacting medical practice.
